# Compliance with infection control practices among healthcare workers in radiology departments: a participant observation study and adenosine triphosphate assay evaluation of environmental cleanliness

**DOI:** 10.1016/j.infpip.2025.100441

**Published:** 2025-02-11

**Authors:** Leonia Hiu Wan Lau, Fion Wai Fong Tse, Lorna Kwai Ping Suen, Simon Ching Lam

**Affiliations:** a School of Nursing, Tung Wah College, Hong Kong Special Administrative Region of China; b School of Nursing, The Hong Kong Polytechnic University, Hong Kong Special Administrative Region of China

**Keywords:** Radiology department, Hand hygiene, Healthcare workers, Standard precautions, Observational study, Adenosine triphosphate assay

## Abstract

**Background:**

Increased risk of healthcare-associated infections (HAIs) in radiology departments has been reported in recent years. Optimal infection control practices (ICPs) are the key to controlling HAIs, but few studies have investigated the ICP compliance of healthcare workers (HCWs) in radiology departments.

**Aim:**

To assess the level of ICP compliance of HCWs in a radiology department.

**Methods:**

A participant observation supplemented with adenosine triphosphate (ATP) bioluminescence assay evaluation of environmental cleanliness was conducted. More than 1000 hand hygiene (HH) opportunities and 960 opportunities for the other core ICP elements were observed in different study units of a radiology department. An online checklist powered by eRub was used to record the HCWs' ICP performance. A total of 125 environmental samples were collected for ATP assay evaluation.

**Results:**

In the participants observation, the overall performance score was 41.4%. The lowest score was found in HH (10.4%), followed by use of personal protective equipment (29.4%) and gloves (68.2%). The HH performance was significantly associated with study units (χ^2^ = 42.55, *P* < 0.001), professional groups (χ^2^ = 110.21, *P* < 0.001), and patient-to-staff ratio (*F* = 8.76, *P* < 0.001). With relative light units of ≤250 set as a pass benchmark, less than two-thirds of the environmental samples passed the ATP assay.

**Conclusion:**

The overall ICP compliance among the HCWs in the radiology department was suboptimal. Revisions of current ICP guidelines and policies that are tailored to the setting-specific needs and barriers in radiology departments is necessary.

## Introduction

The recent COVID-19 pandemic has created a perfect storm for healthcare-associated infections (HAIs) and highlighted the importance of infection control practices (ICPs) in healthcare settings. Infection control practices have long been designed and implemented to prevent the healthcare-associated transmission of infectious agents by interrupting the chain of infection [1]. Transmission of infection within healthcare settings requires the presence of infectious agent, susceptible host, and a favourable environment facilitating the transmission [1]. Healthcare workers (HCWs) are often the well-recognized vectors for HAI transmission, given the high contact rate with patients and high exposure risk to pathogens [2]. Besides, the role of environmental contamination in the infection chain of HAIs has been increasingly reported [3,4]. Cross-transmission of pathogens among patients and HCWs occurs through effective contact (directly or indirectly), or through exposure to contaminated aerosols/droplets. To ensure adequate protection, ICPs currently consist of two tiers: standard and transmission-based precautions. Standard precautions are minimum-level infection prevention practices always applied to all patients, including hand hygiene (HH), use of gloves, use of personal protective equipment (PPE), respiratory hygiene, sharps safety, environmental cleansing and disinfection, and waste disposal [5]. Transmission-based precautions are practices adopted in addition to standard precautions to prevent the spread of epidemiologically important or highly transmissible pathogens (e.g. tuberculosis, influenza, and meticillin-resistant *Staphylococcus aureus*) through specific routes of transmission (e.g. contact, respiratory droplets, airborne spread) [6].

Effective implementation of ICPs could prevent up to 70% of HAIs [7]. However, suboptimal ICP compliance among HCWs has been reported in high- and low-income settings over the years [8,9]. Currently, there is no standardized approach for a comprehensive assessment of ICP compliance. Such an approach is important for informing the development of interventions to boost compliance. Direct observation is considered the gold standard for assessing ICP compliance, which could be performed as participant or non-participant observation [10]. Whereas participant observation involves the observer's participation (as a part of the group being studied for a closer observation with a clear understanding on the group's dynamics and culture), non-participant observation allows a wider observation with the observer remaining outside the group [11]. However, direct observation might suffer from selection bias and the Hawthorne effect [10]. In light of the role of environmental contamination in HAI transmission, evaluation of environmental cleanliness has been proposed to complement direct observation in the assessment of ICP compliance. Several methods for environmental cleanliness evaluation are available, including visual inspection and fluorescent marker (which monitor the cleaning process), as well as microbiological culture and adenosine triphosphate (ATP) bioluminescence assay (which measure the outcome of cleaning) [12].

Radiology departments serve as integral components of the healthcare system, accommodating hundreds of inpatients and outpatients daily for diagnostic, monitoring, and treatment purposes. In recent years, the risk of HAIs in radiology departments has considerably increased [13]. Increased number and variety of patients, frequent and close interactions between patients and HCWs, and high patient rotation in radiology departments greatly facilitate the transmission of pathogens [13]. Furthermore, contamination of high-touch surfaces, radiology equipment, and medical devices in radiology departments has been increasingly reported, representing an important reservoir for HAIs [14,15]. The compliance of HCWs in radiology departments with ICPs has been evaluated in a limited number of studies, but the findings were heterogeneous [16]. Besides, most published studies have focused on self-reported surveys, which might introduce social desirability bias and might not be sufficient to provide an accurate evaluation of ICP compliance [16]. Against such a background, we set out to assess the level of ICP compliance of HCWs in a radiology department through participant observation supplemented with environmental cleanliness evaluation.

## Methods

### Study design and settings

A cross-sectional multi-method study was conducted. The ICP compliance of HCWs in a radiology department was assessed through participant observation supplemented with environmental cleanliness evaluation. This study was conducted in the radiology department of a local 600-bed tertiary care general hospital between January and April 2019. The radiology department being studied was divided to include an intervention area where direct patient contact with HCWs and radiology equipment was likely, nursing station, patient waiting area, and patient transport area. For investigation, the intervention area was further divided into five study units according to the nature of healthcare services provided and geographic location: computed tomography (CT), interventional radiology (IR), magnetic resonance imaging (MRI), ultrasound (US), and X-ray. The Strengthening the Reporting of Observational Studies in Epidemiology (STROBE) guideline for reporting was followed.

### Sampling

All HCWs working in the radiology department, including radiologists, radiographers, nurses, and patient care assistants, were selected through convenience sampling for participant observation. Staff from other departments who presented in the radiology department occasionally, say patient escorts, were excluded. In accordance with the recommendations from the World Health Organization (WHO) on the minimal number of observations required for valid determination of HH compliance, we observed at least 200 opportunities in each study unit [17]. Overall, more than 1000 opportunities were observed in the radiology department. Based on previous experience of the research team, such unit-based observation approach allowed more accurate estimation of ICP compliance and facilitated better comparisons between study units and settings [18,19]. The observation schedule (only weekdays because no service is scheduled at weekends) was constructed on the basis of the routine schedule of each study unit. For instance, IR was scheduled for observation on Thursday and Friday only, whereas MRI was scheduled for observation every Tuesday and Wednesday. A pilot study revealed that 10 opportunities could be observed in each 20 min session. Hence, the designated number of observations could be completed in approximately 10–12 weeks (i.e. 10 opportunities × 2 sessions/week × 10 weeks = 200 opportunities).

Random sampling was adopted in the evaluation of environmental cleanliness. For participant observation, the research assistant (>3 years of working experience) was familiar with the working pattern, group dynamics, and culture. The research assistant observed and recorded the cleaning schedules of each study unit. All the high-touch areas (e.g. A1 = keyboard X in computed tomography) were coded and randomized to generate a sampling list. Our previous work on evaluation of environmental cleanliness indicated that the appropriate sampling time should be just before cleaning [20]. The research assistant complied with the sampling list and sampling time for data collection. Approximately 125 environmental samples (25 samples per study unit) were obtained from the radiology department during the period of observation. A previous study has demonstrated that 125 environmental samples were sufficient to characterize environmental contamination in a healthcare setting [15].

### Participant observation

To gain closer insight into HCWs' ICP performance, participant observation was used. Infection control activities of the HCWs were recorded with an electronic ICP observational checklist powered by eRub (http://www.flowmedik.com; SAG Flowmedik Oy, Finland). eRub has been used by previous observational studies [18,19]. Constructed based on the WHO ‘Five Moments for Hand Hygiene’ and the infection control guidelines laid down by the Centers for Disease Control and Prevention [5,17], eRub ensures the expected behaviour in HH, and other ICP elements were explicitly captured and objectively evaluated. HH activities, including the timing (before and after patient contact, before aseptic task, after body fluid exposure risk, and after contacting patient surroundings) and duration, were comprehensively observed and recorded. Meanwhile, other ICP elements (i.e. glove use; use of PPE; respiratory hygiene; environmental cleaning and disinfection; handling of used linens; decontamination of reusable equipment; sharps safety; and waste management) were observed and rated when opportunities arose. HH performance was rated using a three-point scale (2: well performed (washing hands for ≥20 s with water and soap or rubbing hands for ≥20 s with alcohol-based hand rub); 1: performed but inadequate (<20 s); 0: missed performing) [21]. The performance of other ICP elements was rated using another three-point scale (2: properly performed (performed the described ICP with >80% correctness); 1: improperly performed (performed the described ICP with <80% correctness); 0: missed performing (did not perform the described ICP)) (see Supplementary Appendix for the observational checklist) [21]. eRub demonstrated satisfactory content validity, with the content validity indices determined by six infection control experts ranging from 0.83 to 1.00. Selection bias and Hawthorne effect were minimized by employing a staff nurse of the studied radiology department as the observer, who was rostered to have the observation performed at random time-slots covering morning, afternoon, and night shifts [22]. Furthermore, the observer was trained and validated to have the ICP knowledge and skills required to observe behavior in a neutral and non-judgemental manner [23].

### Measurement: evaluation of environmental cleanliness

Environmental cleanliness was evaluated using a 3M clean-trace hygiene monitoring and management system (3M Corp, Hong Kong) called the adenosine triphosphate (ATP) bioluminescence assay. ATP bioluminescence assays detect and quantify the amount of ATP from living micro-organisms and organic debris present on environmental surfaces. ATP bioluminescence assays cannot replace classic microbiological culture in the measurement of environmental microbial contamination, yet it serves a unique role in the context of ICP compliance assessment. With an appropriate benchmark value selected, the ATP bioluminescence assay provides a real-time surrogate of the efficacy of environmental cleaning in reducing microbial contamination, allowing rapid determination of environmental cleanliness and ICP compliance [[24], [25], [26]]. According to the manufacturer's instructions, environmental samples were collected by swapping 10 × 10 cm^2^ (4 in. × 4 in.) area of the different touch points of the high-touch surfaces, radiology equipment, and medical devices in each study unit randomly. The ATP level in the sample was measured using a luminometer and was expressed as bioluminescence relative light units (RLU). A clean threshold of ≤250 for RLU was adopted as a pass benchmark in this study [27]. Our team had track records for data collection by the use of ATP elsewhere [20].

### Data analysis

The infection control performance of HCWs in the participant observation was presented as performance scores, and results from ATP bioluminescence assay in the environmental cleanliness evaluation were summarized as ATP pass rate, which altogether reflected the degree of ICP compliance. Performance score for each ICP element was calculated as the proportion of the total number of opportunities observed in which well/properly performed ICP activities were taken. The ATP pass rate was calculated as the proportion of environmental sample with ≤250 RLU. χ^2^-Test and one-way analysis of variance were used in inferring the association between ICP compliance with study units, professional groups, and workforce factors. Data were analysed using SPSS®-PC version 25 with statistical significance defined at *P* < 0.05.

## Results

### Results of participant observation

During the 11-week study period, a total of 2078 ICP opportunities were observed. The ICP elements observed included HH (*N* = 1110), glove use (*N* = 617), use of PPE (*N* = 17), respiratory hygiene (*N* = 118), environmental cleaning and disinfection (*N* = 28), decontamination of reusable equipment and handling of used linen (*N* = 39), waste management (*N* = 83), and sharps safety (*N* = 66) (Table I). The overall ICP performance score was 41.4%. In particular, the performance scores were 10.4%, 68.2%, 29.4%, 96.6%, 96.4%, 79.5%, 100%, and 98.5%, for HH, glove use, use of PPE, respiratory hygiene, environmental cleansing and disinfection, decontamination of reusable equipment and handling of used linen, waste management, and sharps safety respectively (Table I).

**Table I:** Number of observed infection control practice (ICP) opportunities and performance of participants

No. of observed ICP opportunities	ICP elements								
	All ICPs	Hand hygiene	Glove use	Use of PPE	Respiratory hygiene	Environmental cleansing and disinfection	Decontamination of reusable equipment and handling of used linen	Waste management	Sharps safety
Total no.	2078	1110	617	17	118	28	39	83	66
Well performed/properly performed	861 (41.4%)	115 (10.4%)	421 (68.2%)	5 (29.4%)	114 (96.6%)	27 (96.4%)	31 (79.5%)	83 (100%)	65 (98.5%)
Performed but inadequate/improperly performed	525 (25.3%)	332 (29.9%)	190 (30.8%)	0	0	0	2 (5.1%)	0	1 (1.5%)
Missed performing	692 (33.3%)	663 (59.7%)	6 (0.9%)	12 (70.6%)	4 (3.4%)	1 (3.6%)	6 (15.4%)	0	0

ICP, infection control practice; PPE, personal protective equipment (including grown, face shield and mask).

**Figure 1:**
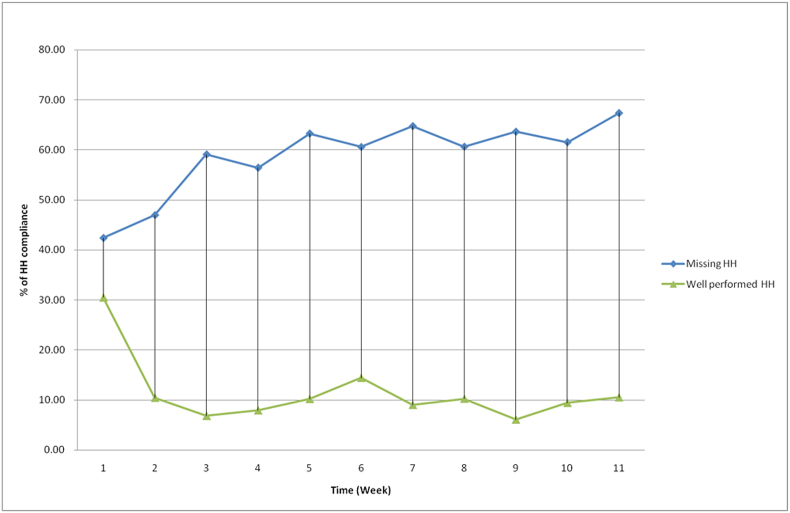
Temporal changes of perform score of hand hygiene practices across the 11-week study period.

HH performance was significantly different among the Five Moments of indication (χ^2^ = 435.01, *P* < 0.001) (Table II). The most frequently observed moments of indication for HH were ‘before patient contact’ and ‘after patient contact’. However, the rate of well-performed HH was only 1.7% and 11.8%, and HH was missed in ∼94% and ∼45% of the opportunities during the moment ‘before patient contact’ and ‘after patient contact’, respectively. Enhanced HH performance was observed ‘before aseptic tasks’, in which well-performed HH was demonstrated in more than half (57.6%) of the HH opportunities. Significant differences in HH performance were found between professional groups (χ^2^ = 110.21, *P* < 0.001) and study units (χ^2^ = 42.55, *P* < 0.001) (Table II). Whereas participants in the IR unit demonstrated well-performed HH in 20.2% of the opportunities, the rates of well-performed HH were <10% in the CT, MRI, US, and X-ray units. Among the four professional groups, radiologists and nurses had significantly higher HH performance score as compared with radiographers and healthcare assistants (27.6% for radiologists and 14.7% for nurses vs 6.7% for radiographers and 5.6% for healthcare assistants). Temporal changes in HH performance over the 11-week study period are illustrated in Figure 1. The rate of well-performed HH was as high as 30% during the first week, which sharply decreased in the second week and stabilized at ∼10% in the remaining weeks. Regarding the relationship between HH performance and workload factors, better HH performance was significantly associated with decreased number of patients attended (*F* = 9.54, *P* < 0.001) and increased patient-to-staff ratio (*F* = 8.76, *P* < 0.001) (Table III). In handling bed-bound patients or patients under airborne, droplet, or contact precaution, HCWs failed to perform HH in nearly one-third (38.7%) of the opportunities. HH was missed in 96.3% of the opportunities during the moment ‘before patient contact’ but only 6.8% ‘after patient contact’ (Supplementary Table S1).

**Table II:** Performance of hand hygiene practices by WHO Five Moments of indication, study units and professional groups

	Observed hand hygiene opportunities				χ^2^-Test
	Total (*N* = 1110)	Missing hand hygiene (*n* = 663)	Performed but inadequate (*n* = 332)	Well performed (*n* = 115)	
WHO Five Moments of indication for hand hygiene^a^					
Before patient contact	421	394 (93.6%)	20 (4.8%)	7 (1.7%)	χ^2^ = 435.01 *P* < 0.001
After patient contact	558	253 (45.3%)	239 (42.8%)	66 (11.8%)	
Before aseptic task	33	4 (12.1%)	10 (30.3%)	19 (57.6%)	
After body fluid exposure risk	73	4 (5.5%)	49 (67.1%)	20 (27.4%)	
Patient surroundings	25	8 (32%)	14 (56%)	3 (12%)	
Study units^a^					
Computed tomography	224	145 (64.7%)	61 (27.2%)	18 (8.0%)	χ^2^ = 42.55 *P* < 0.001
Interventional radiology	223	104 (46.6%)	74 (33.2%)	45 (20.2%)	
Magnetic resonance imaging	221	134 (60.6%)	69 (31.2%)	18 (8.1%)	
Ultrasound	217	120 (55.3%)	77 (35.5%)	20 (9.2%)	
X-ray	229	160 (69.9%)	51 (22.3%)	18 (7.9%)	
Type of occupation					
Radiologist	127	57 (44.9%)	35 (27.6%)	35 (27.6%)	χ^2^ = 110.21 *P* < 0.001
Nurse	265	107 (40.4%)	119 (44.9%)	39 (14.7%)	
Radiographer	421	289 (68.6%)	104 (24.7%)	28 (6.7%)	

WHO, World Health Organization.

**Table III:** Relationship between hand hygiene performance and workforce factors

Workforce factors	Observed hand hygiene opportunities				ANOVA
	Total (*N* = 1110)	Missing hand hygiene (*n* = 663)	Performed but inadequate (*n* = 332)	Well performed (*n* = 115)	
No. (SD) of patients attended	–	21.9 (20.6)	17 (17.5)	15.8 (18.2)	*F* = 9.54, *P <* 0.001
No. (SD) of staff on duty	–	3.6 (0.5)	3.6 (0.5)	3.5 (0.6)	*F* = 2.46, *P =* 0.086
Patient-to-staff ratio (SD)	–	6.1 (5.9)	4.8 (4.9)	4.5 (5.0)	*F* = 8.76, *P <* 0.001

SD, standard deviation; ANOVA, analysis of variance.

In nearly two-thirds (68.2%) of opportunities, participants demonstrated proper use of gloves (Table I). The improper use of gloves was observed in 30.8% of the opportunities. Most participants failed to perform HH after glove removal. Some of them used the same gloves while providing services to different patients, wore multiple gloves, or took off the outer gloves only after providing services to patients. Failure to wear gloves during the procedures that exposed them to body fluids, secretion, blood, excretion, non-intact skin, or mucous membranes was observed in only 1% of the opportunities. Significant difference in the performance of glove use was found among professional groups (χ^2^ = 314.84, *P* < 0.001) (Table IV). However, the four cells (20.0%) had an expected count of <5. Hence, the analytical result should be interpreted with caution.

**Table IV:** Performance of glove use by professional group

Professional groups	Observed glove use opportunities				χ^2^-Test
	Total (*N* = 627)	Properly used (*n* = 663)	Improperly used (*n* = 332)	Not used (*n* = 115)	
Radiologist	109	103 (94.5%)	6 (5.5%)	0	χ^2^ = 314.84, *P* < 0.001
Nurse	196	146 (74.5%)	48 (24.5%)	2 (1.0%)	
Radiographer	149	96 (64.4%)	50 (33.6%)	3 (2.0%)	
Healthcare assistant	163	76 (46.6%)	86 (52.8%)	1 (0.6%)	

### Results of environmental cleanliness evaluation

Among the 125 environmental samples collected for ATP bioluminescence assay, samples from the CT unit were considered the most contaminated. Only 26.1% of these samples were regarded as clean (≤250 RLU). The samples from the US unit were the least contaminated (81.5% were clean). The samples from the IR, MRI, and X-ray unit yielded similar results. Significant difference in ATP pass rate was observed among the units (χ^2^ = 17.36, *P* = 0.002) (Table V).

**Table V:** Evaluation of environmental cleanliness: adenosine triphosphate passing rate by study units

	Units						χ^2^-Test
	Total	CT	IR	MRI	US	X-ray	
Environmental samples obtained	125	23	22	26	27	27	
Passed^a^	77 (61.6%)	6 (26.1%)	15 (68.2%)	17 (65.4%)	22 (81.5%)	17 (63.0%)	χ^2^ = 17.36, *P* = 0.002
Failed	48 (38.4%)	17 (73.9%)	7 (31.8%)	9 (34.6%)	5 (18.5%)	10 (37.0%)	

CT, computed tomography; IR, interventional radiology; MRI, magnetic resonance imaging; US, ultrasound.

a A clean threshold of relative light units (RLU) ≤250 was adopted as a pass benchmark.

## Discussion

To the best of our knowledge, this study was the first to employ participant observation supplemented with environmental cleanliness evaluation to assess ICP compliance in HCWs in a radiology department. Furthermore, this study was conducted one year before the COVID-19 outbreak and hence could serve as a baseline for comparing pre- and post-COVID-19 ICP performance. In the participant observation, the overall performance score was low (41.4%). The performance score varied among different ICP elements, with the lowest score found in HH (10.4%), followed by the use of PPE (29.4%) and use of gloves (68.2%). Significant difference in HH performance was found among the five moments of indication, study units, and professional groups. HH performance was significantly associated with workload factors, such as patient-to-staff ratio. In the evaluation of environmental cleanliness, less than two-thirds of environmental samples passed the ATP test. Given the expansion of scope of services and the increased risks of HAIs in radiology departments in recent years, the study findings were worrisome.

The HH compliance of HCWs in the radiology department was unacceptably low, with the performance score of only 10.4%. An overall HH compliance of 74.7% was reported by a local study that performed 1037 observations in four clinical settings (medical wards, surgical wards, accident and emergency departments, and intensive care units) in acute hospitals and two clinical settings (medical and surgical) in two rehabilitation hospitals [23]. Another local study showed HH compliance rates of 79.8–100% throughout 380 observations in a paediatric setting [28]. A recent participant-observational study conducted in a psychiatric hospital setting through closed-circuit televisions (with more than 2000 observations) showed HH compliance rate of 3.3% [18]. Methodological issues (e.g. observation approach, sampling strategies, definition regarding the hand-hygiene opportunities and compliance to be observed, unavoidable Hawthorne effect) could be one of the reasons contributing to the discrepancy in findings [10,22]. On the other hand, the large discrepancy in HH compliance between settings might suggest that a ‘one size fits all’ approach is not sufficient for achieving optimal HH compliance [29]. Such approaches fail to acknowledge the local circumstances and the unique challenges at the individual (e.g. knowledge, attitude, decision process), interpersonal (peer influence, professional role and identity), and institutional (workload, ward setting, accessibility of HH facilitates and product) levels. Our study found that ‘before patient contact’ was the moment with the poorest HH performance (missed HH: 93.6%; well-performed HH: 1.7%), and there was higher likelihood for the HCWs to practice HH ‘after patient contact’ and ‘after body fluid exposure risk’. These findings were consistent with Eckmanns *et al.*‘s and previous local observational studies, suggesting that self-protection and risk perception are the major motivations of HH [18,19,30]. In addition, HCWs' HH performance appeared to have improved (HH performed in 93.2% and 100% of opportunities ‘after patient contact’ and ‘after body fluid exposure risk’ respectively) when encountering high-risk cases (i.e. bed-bound patients or patients under airborne, droplet, or contact precautions). The finding aligned with that of Kuzu and White *et al.*, further supporting that HCWs practised HH as a means of self-protection rather than patient protection [31,32]. Well-performed HH was observed in more than half of the opportunities (57.6%) ‘before aseptic task’. Professional identity, which is a perception focusing not only on self-oriented values (e.g. self-protection and workload) but also profession-oriented values (i.e. expected actions of HCWs on behalf of patients' well-being), could be the underlying reason [33,34]. The HH compliance was generally higher in radiologists and nurses than in radiographers and supporting workers. These observations might be related in part to the cohort effect and difference in scope of work among professional groups [35]. Radiologists and nurses were more likely to participate in high-risk and invasive procedures, which might increase their own risk perception and thus HH compliance.

HH practice is a complex behaviour. Beyond the inherent component driven primarily by self-protection, HH includes an elective component, which could be confronted with competing priorities [36]. This might help explain the significant association between HH performance and workload factors, such as patient-to-staff ratio. High patient-to-staff ratio and time limitation have been identified as important organizational barriers to HH adoption [37,38]. A study explaining HH beliefs and practices in relation to the working environment suggested that dealing with emergency situations, being extremely busy, being distracted, and having insufficient time were major barriers to HH [39]. The reinforcement of the elective component of HH should be the focus in an HH promotion programme. This, coupled with auditing and performance feedback, education and practical training tailored to the characteristics and needs of each professional group in the radiology setting could be effective [40]. Promoting professional identity, creating institutional safety climate, providing sufficient hand hygiene supplies, and having reminders in workplace might also support the implementation of HH [41]. Furthermore, the current HH protocol was developed on the basis of the WHO Five Moments for Hand Hygiene. However, the direct application of the Five Moments was challenged by the ‘inconvenient truths’ (e.g. not always possible to implement Five Moments for all patients all the time; the patient zone is not a fixed entity; barriers that reduce HH adherence were overlooked), particularly in settings where distinguishing between patient and healthcare zone is difficult (e.g. radiology departments) [42]. The current HH protocol should be revised. It would be effective to augment the Five Moments with the unique characteristics of settings, setting-specific HAI risk, and HCWs' contemporary needs considered [42].

The appropriate use of gloves was demonstrated in more than two-thirds of the opportunities (68.2%). Notably, some HCWs did not change gloves between caring for different patients. Some HCWs were observed wearing multiple pairs of gloves and removing the outer pair each time after patient care. The overuse of gloves was not uncommon. HCWs wore gloves during procedures in which risk of contact with blood, body fluids, and excretion was non-existent. Continuous wearing of gloves and omitting HH after glove removal was observed as well. The improper use of gloves was found to be driven by emotions, such as fear and disgust, socialization within the profession, and the misconception that gloves could be a substitute for HH [43]. The omission of PPE was found in more than two-thirds of the opportunities (68.2%). Failure to identify appropriate timing for PPE donning and low risk perception associated with transient contact with patients in the radiology department could be the underlying reasons. In infection control, PPE and gloves are used as a barrier between infective pathogens and HCWs, and their improper use might in turn increase the risk of HAI transmission. Hence, education and guidance are still needed to promote proper PPE and glove use in radiology departments.

Environmental cleaning is an important pillar of infection control in radiology departments, yet less than two-thirds of environmental samples in the studied radiology department passed the ATP test. The study findings probably reflected suboptimal ICP compliance and the poor implementation of environmental cleaning practices. However, the performance score of environmental cleaning and disinfection was high in the participant observation. The ATP passing rate varied among the five study units, in which the US unit was the least contaminated (81.5%), followed by the IR (68.2%), MRI (65.4%), X-ray (63%), and CT (26.1%) unit. The discrepancies among findings, together with the large variation in ATP passing rate among the five study units, suggested that proper environmental cleaning in radiology departments could be complicated by structural features, the number and type of patients attending, and contact pattern with patients in different study units. Furthermore, the presence of biofilms and associated adaption or resistance to disinfectants could greatly decrease the effectiveness of environmental cleaning despite the appropriate implementation of the guideline [44]. The enhancement of current environmental cleaning guidelines by defining surfaces with different levels of contamination (i.e. high, moderate, and low) coupled with evidence-based recommendations regarding the selection and dilution of disinfectants, techniques of cleansing, and duration and frequency of cleaning practices would be necessary [45].

Our study was not without limitations. First, although we adopted the WHO recommendation to observe at least 200 opportunities for each study unit, only a limited number of opportunities were observed for some ICP elements, such as the use of PPE, environmental cleaning and disinfection, and decontamination of reusable equipment and handling of used linen (17–39 opportunities). Thus, the result may not be sufficient to reflect the true compliance of HCWs to these ICP elements. Second, an ATP bioluminescence assay was used in evaluating environmental cleanliness in this study. Given the weak correlation between ATP measurement and level of microbial burden, and that the choice of pass benchmark could affect the test's sensitivity and specificity, the findings should be interpreted with caution. Supplementing ATP bioluminescence assay with microbial culture and antimicrobial susceptibility in future studies could allow better capture of the environmental cleanliness. Third, the quantitative approach employed in this study limited the qualitative inquiry to explain the ICP behaviour of the participants. Lastly, the use of the participant-observation approach might be subject to the Hawthorne effect and observer bias. Nevertheless, our study provided comprehensive evidence of ICP compliance in HCWs in radiology departments. Future studies should explore the individual's decision-making process regarding ICP implementation in radiology settings. Considerable effort should be placed on revising current ICP guidelines and tailoring them according to the unique characteristics of radiology settings, setting-specific HAI risk, and HCWs' risk perception and contemporary needs.

## Author contributions

Conceptualization: F.W.F.T. and S.C.L.; methodology; F.W.F.T. and S.C.L.; writing – original draft: F.W.F.T and S.C.L.: writing – review and editing: L.H.W.L. and L.K.P.S.; project administration: F.W.F.T. and S.C.L.

## Ethics statement

Ethical approval was obtained from the Research Ethics Committee of Hospital Authority (REC Ref. No.: HKECREC-2019-008), Human Subjects Ethics Application Review System of the Hong Kong Polytechnic University (Ref No.: HSEARS20190112001), and the chief-of-service and manager of the radiology department. Information sheets regarding the study purpose and data collection procedure were provided to all the participants during an internal conference meeting. Informed consent was obtained from the participants and the respective ward manager before data collection, and confidentiality and voluntary participation were emphasized. However, the time when they would be observed was not disclosed.

## Data availability

The data and materials are available from the corresponding author upon reasonable request.

## Funding sources

This project is partially supported by the College Research Grant (Ref: CRG2022/06), Tung Wah College and the Research Matching Grant Scheme (Ref: RMGS220403), University Grants Committee, 10.13039/501100017649The HKSAR Government.

## Conflict of interest statement

The corresponding author is an Editor for *Infection Prevention in Practice*. Editorial policies were followed to ensure editorial independence, and the manuscript was handled by separate Editors.
