# Supplementary material for: Compliance with infection control practices among healthcare workers in radiology departments: a participant observation study and adenosine triphosphate assay evaluation of environmental cleanliness
Source: Infect Prev Pract. 2025 Feb 11;7(2):100441. doi: 10.1016/j.infpip.2025.100441 (PMC11910355; doi:10.1016/j.infpip.2025.100441)
Supplement: Multimedia component 1 [file mmc1.docx]

**Infection Prevention in Practice (IPIP)**

**TITLE: Compliance with infection control practices among healthcare workers in radiology departments: a participant observation study and adenosine triphosphate assay evaluation of environmental cleanliness**

**Leonia Hiu Wan LAU ^1#^, Fion Wai Fong TSE^2#^, Lorna Kwai Ping SUEN ^1^, Simon Ching LAM ^1*^**

1. School of Nursing, Tung Wah College, Hong Kong SAR, China.
2. School of Nursing, The Hong Kong Polytechnic University, Hung Hom, Hong Kong SAR, China.

**Supplementary materials**

**Supplementary Table I. Hand hygiene performance in handling of bed-bound (relatively weak) cases / cases with infectious precautions**

|  | **Moment of indications for hand hygiene** | | | | | |
| --- | --- | --- | --- | --- | --- | --- |
|  | **Total** | **Before patient contact** | **After patient contact** | **Before aseptic task** | **After body fluid exposure risk** | **After contacting patient surroundings** |
| Observed HH opportunities | 104 | 27 | 44 | 1 | 2 | 1 |
| Missing hand hygiene, n (%) | 29 (38.7%) | 26 (96.3%) | 3 (6.8%) | 0 (0%) | 0 (0%) | 0 (0%) |
| Performed hand hygiene, n (%) | 75 (61.3%) | 1 (3.7%) | 41 (93.2%) | 1 (100%) | 2 (100%) | 1 (100%) |

n = number of observed hand hygiene opportunities

% (column percentage) = the proportion of observed opportunities in which the hand hygiene was performed and missed performing respectively.

**Infection control practice observational checklist powered by eRub** (digital service provided by SAG Flowmedik Oy, <https://www.flowmedik.com/en/>)

**Section 1: Demographics**

Unit being observed: ___________

Profession^*^: Allied health professionals / Doctors / Nurses / Health workers / Personal Care workers

**Section 2: Five moments of hand hygiene**

| **Items** | **Hand disinfectant usage time is recorded (in seconds) ^** |
| --- | --- |
| 1. Hand hygiene performed before patient contact |  |
| 2. Hand hygiene performed after patient contact |  |
| 3. Hand hygiene performed before aseptic task ... |  |
| 4. Hand hygiene performed after body fluid exposure risk |  |
| 5. Hand hygiene performed after contact with patient surroundings |  |
| 6. Hand disinfectant dispenser near: Yes / No | |

Remarks: ^0 second means missing the hand hygiene practice

**Section 3: Other Practice of Standard Precautions^#^**

| **Items** | **Not Done** | **Improperly done** | **Properly done** | **Not applicable** |
| --- | --- | --- | --- | --- |
| 7. Use of personal protective equipment (grown, face shield and mask) | 0 | 1 | 2 | NA |
| 8. Respiratory hygiene | 0 | 1 | 2 | NA |
| 9. Handling sharp equipment (sharp safety) | 0 | 1 | 2 | NA |
| 10. Environmental cleansing and disinfection | 0 | 1 | 2 | NA |
| 11. Clinical waste disposal | 0 | 1 | 2 | NA |
| 12. Decontamination of reusable equipment & handling of used linen | 0 | 1 | 2 | NA |

13. Add risk factor observation:

🞏 Ring / 🞏 Wristband or watch / 🞏 Artificial nails / 🞏 Long nails / 🞏 Nail polish

🞏 Other ____________________

14. Add observation of gloves:

🞏 No gloves used / 🞏 Gloves used correctly / 🞏 Gloves used incorrectly

Remarks:

^*^Profession:

Allied Health professionals = Physiotherapist, Occupational Therapist, Radiographer, Speech therapist, and other appropriate.

Doctors = Licensed medical practitioners including traditional Chinese Medicine practitioners, and medical students.

Nurses = Licensed nursing professionals and nursing students.

Health workers = Assistants with certificates or in training, e.g., Health Care Assistants, Physiotherapists Assistants or Occupational therapists Assistants or Radiographers Assistants.

Personal Care Workers = Provision of direct nursing and patient care with training (but without a certificate), tasks mainly on client’s personal and household hygiene, meal preparation and etc.

^#^ Other Practice of Standard Precautions:

Not done (0) = missed performing (did not perform the described infection control practice)

Improperly done (1) = improperly performed (performed the described infection control practice with less than 80% correctness)

Properly done (2) = properly performed (performed the described infection control practice with over 80% correctness)

Not applicable (NA) = not applicable for the observed situation

^#^Decision between “Not done (0)”, “Improperly done (1)” Properly done (2)” and “Not applicable (NA)” is based on the infection control guidelines from Centers for Disease Control and Prevention, and Hospital Authority & Department of Health HKSAR.

Centers for Disease Control and Prevention. (2024). Infection Control Basics. <https://www.cdc.gov/infection-control/hcp/basics/index.html>

Scientific Committee on Infection Control of Department of Health, Task Force on Infection Control of Hospital Authority. (2019). Recommendations on Implementing Isolation Precautions in Hospital Settings. <https://www.chp.gov.hk/files/pdf/recommendations_on_implementing_isolation_precautions_in_hospital_settings.pdf>

----------------------------------------------------------------------------------------------------------------------

The Corresponding Author (Prof. Simon C. LAM) retains the copyright of the above eRub checklist.
